# The elderly in the psychiatric emergency service (PES); a descriptive study

**DOI:** 10.1186/1471-244X-11-111

**Published:** 2011-07-15

**Authors:** Yves Chaput, Lucie Beaulieu, Michel Paradis, Edith Labonté

**Affiliations:** 1 365, Rue Normand, suite 230, Saint-Jean-sur-Richelieu J3A 1T6, Quebec, Canada; 2 Department of Psychiatry, Haut-Richelieu Hospital, 920 Boulevard du Séminaire Nord, Saint-Jean-sur-Richelieu J3A 1B7, Quebec, Canada; 3 Department of Psychiatry, Notre-Dame Hospital, 1560 Sherbrooke street East, Montreal H2L 4M1, Quebec, Canada; 4 Department of Psychiatry, Enfant-Jesus Hospital, 1401 18th street, Quebec G1J 1Z4, Quebec, Canada

## Abstract

**Background:**

The impact of an aging population on the psychiatric emergency service (PES) has not been fully ascertained. Cognitive dysfunctions aside, many DSM-IV disorders may have a lower prevalence in the elderly, who appear to be underrepresented in the PES. We therefore attempted to more precisely assess their patterns of PES use and their clinical and demographic characteristics.

**Methods:**

Close to 30,000 visits to a general hospital PES (Montreal, Quebec, Canada) were acquired between 1990 and 2004 and pooled with over 17,000 visits acquired using the same methodology at three other services in Quebec between 2002 and 2004.

**Results:**

The median age of PES patients increased over time. However, the proportion of yearly visits attributable to the elderly (compared to those under 65) showed no consistent increase during the observation period. The pattern of return visits (two to three, four to ten, eleven or more) did not differ from that of patients under 65, although the latter made a greater number of total return visits per patient. The elderly were more often women (62%), widowed (28%), came to the PES accompanied (42%) and reported « illness » as an important stressor (29%). About 39% were referred for depression or anxiety. They were less violent (10%) upon their arrival. Affective disorders predominated in the diagnostic profile, they were less co-morbid and more likely admitted than patients under 65.

**Conclusion:**

Although no proportional increase in PES use over time was found the elderly do possess distinct characteristics potentially useful in PES resource planning so as to better serve this increasingly important segment of the general population.

## Background

The median age of Canada's population has been increasing since 1966, with those aged 14 and under declining since 1996, attaining 17% of the population (their lowest level in 2006) versus 13.7% for those 65 and over (≥ 65) [1]. The impact of an aging population on major service points in mental health care delivery, such as the psychiatric emergency service (PES), has yet to be fully ascertained. With the exception of cognitive disorders (CD) and minor or non-specific psychiatric diagnoses, many DSM-IV disorders appear to have a lower prevalence in the elderly [2-7]. A 'Canadian Community Health Survey' suggested a relationship between mental illness severity and the probability of seeking psychiatric help [8]. Given this relationship it is possible that the elderly might less frequently visit the PES for minor psychiatric symptoms. With few exceptions [9], most studies assessing PES use by the elderly in Canada and in the United-States suggest that they are underrepresented in the overall PES population [10-13]. Furthermore, increases in PES use over time by the elderly have yet to be reported.

The elderly with severe mental illness are less likely to visit the psychiatric emergency room than their younger cohorts, making preferential use of mobile psychiatric emergency teams or case-management [13-15]. In addition, even the elderly with major depression are less likely to seek or to receive psychiatric care than those under 65 (< 65) [16-18].

In the general population the most frequent DSM-IV diagnoses (other than CDs) in the elderly are the various anxiety disorders and dysthymia (or minor depression) [2-4,17,19]. In the medical emergency department (ED) anxiety disorders appear to be an important part of the diagnostic profile of the 53 million visits in the United-States (1992 to 2001) for reasons of mental health and, the elderly account for an increasing proportion of these visits [20]. In contrast to their increasing importance in the ED, anxiety disorders contribute little to the typical PES diagnostic profile [10,21-23]. Preliminary evidence however does suggests that the elderly may possess a PES diagnostic profile different from that of patients < 65, one weighted towards cognitive and/or mood disorders [11,24-28], while that of those < 65 is primarily characterized by chronic psychosis, personality and substance abuse disorders [10,21-23]. In addition, factors other than diagnosis may help differentiate the elderly PES user from those < 65. Gender differences (a higher proportion of women), fewer self referrals, a higher hospitalization rate following the visit and the frequent presence of a contributing medical condition, have all been reported [11-13,24,28]. As such, assessing diagnostic, demographic and patterns of PES use by the elderly may contribute to better defining the role and structure of future geropsychiatric emergency health care delivery.

This study had several objectives. Our primary aim was the longitudinal assessment of yearly PES use by the elderly, using a greater than 14-year observation period during which the surrounding PES population was rapidly aging. Second, to assess the patterns of PES use by the elderly, such as multiple visits, over this same time period. Third, it was to add to the preliminary but growing body of clinical and demographic data concerning PES visits made by the elderly. The latter was done using a prospectively acquired database of visits to four PES sites in the province of Quebec, Canada, where each individual visit could contain up to 70 variables.

## Methods

Data collection was as previously described [10,29]. Clinical and demographic data were obtained from all patients 18 years of age and older visiting a major downtown Montreal university teaching hospital PES (the main site) from June 15, 1985, to December 31, 2000. Each PES in metropolitan Montreal is assigned a geographic catchment area and citizens within it are obliged to seek acute psychiatric care at that service only. Approximately 4.8% of all patients who underwent triage in the ED were referred for a psychiatric assessment.

The database began June 15, 1985 as an 'in-house' register kept by the nursing staff. By 1990 it contained eight variables (name, sex, age, catchment area, referral source, date and time of entry to, and departure from the PES, and patient disposition). For research purposes, in July 1996 the database had expanded to include a maximum of 70 variables per visit and was transformed into electronic format, including all data prior to this date. The main table contained administrative variables (chart number, name, sex, and so forth). Linked tables contained variables pertinent to the consultation process, such as date and time of arrival, reasons for the referral, disposition, and so forth. Data entry was performed by designated members of the nursing staff and by the principal investigator. If neither was present, charts were held in the PES until they could be reviewed for all pertinent information. This database was used at the main site from July 1996 to December 2000, after which only the original 8 variables of the register were acquired until September 2002.

The expanded database described above was once again used for a two-year period beginning September 2002, at the main site and at three functionally dissimilar services. Two of these latter services were in cities other than Montreal. One was in Quebec City (300 km east of Montreal, approximately 500,000 citizens) and the other in Saint-Jean-sur-Richelieu (40 km south of Montreal, metropolitan population of approximately 90,000). This latter site differed from the other three by not having an observation area with short-term beds [30]. The second Montreal PES was in a psychiatric institute and did not have an ED (or prior medical triage). As such, it largely functioned as a "walk-in clinic". All variables in the database were listed in a paper format, which was used as the primary triage instrument for all patients visiting the four services during the two-year period. The completed forms were forwarded to the principal investigator on a weekly basis for data entry.

Many strategies were used in order to minimize diagnostic uncertainty in both data collection efforts. First, as over 60% of PES visits have been shown to occur within the daytime hours [10] only services that were covered on weekdays by experienced, regular daytime psychiatric staff with over 5 years experience in the PES setting were included. None of the four sites provided midnight to 7 am assessments. Patients referred to the PES during this time period were kept in the psychiatric observation area for assessment in the morning. As such, during weekdays, well over 70 to 80% of patients were assessed by the regular PES staff. Most staff obtained their medical and specialty training at one of the four medical faculties in the province and thus shared a common set of methodological/ethical/cultural standards. Second, diagnoses, made using DSM-IV guidelines during non-structured clinical interviews, were obtained either directly from staff after the patient assessment or from the patient's chart. Third, diagnoses were grouped into broad categories, which included 'none', 'adjustment', 'anxiety', 'personality', 'affective', 'schizophrenia', 'psychosis not otherwise specified', 'substance abuse' and 'organic mental disorders'. Fourth, in patients with two or more visits a "most probable" primary diagnosis was attributed, which was the diagnosis most frequently given. The second most frequently attributed diagnosis was retained as co-morbidity. Fifth, as previously reported [10], from 65 to 80% of frequent users at all sites were at one point in time under multidisciplinary outpatient care and as such any diagnostic uncertainty could be clarified with the treating team.

### Primary data analysis

Only visits where age could be accurately determined (98% of all visits) were used. Data was analyzed using Systat (Version 13). Three datasets were extracted from the main database. Longitudinal, frequency of PES use and temporal variables were analyzed using data collected between January 1990 and August 2004 at the main site ("A" dataset, 15,579 patients, 29,985 visits). Statistical differences between numerical means (ages, number of visits or time of presentation) for patients 65 and over (versus patients under 65) were determined using *t*-test for non-paired values.

Some clinical variables (diagnoses, pertinence of the visit) were acquired at the main site from July 1996 to December 2000 and similarly acquired at all sites from September 2002 to August 2004. These data were pooled and comprised dataset "B" (21,732 patients, 36,776 visits). Lastly, some socio demographic and clinical variables were only acquired during the multicenter (September 2002 to August 2004) part of this study (dataset "C", 14,850 patients, 22,881 visits, combining all 4 sites).

Most variables of the B and C datasets were of the nominal type (binary or with multiple categories). Preliminary analyses consisted of constructing contingency tables where the binary independent grouping variable (≥ 65, < 65) was tabulated with a response variable (presence or absence of violence...). If the *p*-values for the Pearson chi-square and the likelihood ratio chi-square were < 0.05 then data were re tabulated using a goodness of fit model to determine if the response variable's profile differed significantly from the independent variable using the distribution ratios of the ≥ 65 group as the expected frequencies. If the Pearson and likelihood ratio chi-square *p*-values remained < 0.05 then data were tabulated using multi-way standardized tables with gender as a strata variable. Only data where all three procedures were significant are presented in the results section as "Pchi2 & LRc2, *p *< 0.05". Determining the strength (or direction) of an association between the response and independent variable was not the primary goal of this study. However, when pertinent, logistic regression with the resulting Odds Ratios (uncorrected for gender) and their 95% confidence intervals was used in order to assess it.

This study was approved by the institutional review board (IRB) scientific subcommittees at all sites and was exempted from full review other than at the second Montreal site, where full IRB approval was required and obtained.

## Results

Longitudinal data, temporal patterns and frequency of PES use at the main site.

Patients ≥ 65 represented 8.7% (N = 1349) of all patients and 7.2% of all visits (N = 2171) of the A dataset at our main site. Averaging the Montreal city 1996 and 2001 census the elderly accounted for approximately 14.6% of the city population [1,31]. Of the 1349 patients ≥ 65, 36%, 27%, 20% and 16% were aged between 65 and 69, 70 and 74, 75 and 79 and 80 and over, respectively. Overall, 62% were women (N = 839) versus 44% (N = 6315 of 14,230 patients) of those < 65. During the 14.5-year data acquisition period there was a yearly increase in the mean (from 36.6 ± 18 to 40.8 ± 14) and median (from 35 to 39) age of individual patients (each counted only once/year) visiting this PES. Significant differences were found (p < 0.01, *t*-test for unpaired values) when comparing the average mean age for the first 4 years (38.7 ± 16.7) to that of the last 4 years of the observation period (41.1 ± 14.3). On a yearly basis the mean age of women was always slightly higher than that of men and, in all but the final year (2004) this difference was statistically significant (p < 0.01, *t*-test for unpaired values). The proportion of the total number of yearly visits attributable to patients ≥ 65 (compared to those < 65) showed no consistent increase over time (table 1). When patients/year was analyzed there was a significant reduction in the average proportion of patients ≥ 65 during the last 4 years compared to the first 4 years (7.7 ± .44% versus 9.6 ± .9%, p < 0.05, *t*-test for unpaired values) of this study.

**Table 1:** Proportion (in %) of the total yearly number of visits (V) attributable to patients ≥ 65.

*Year*	*90*	*91*	*92*	*93*	*94*	*95*	*96*	*97*	*98*	*99*	*00*	*01*	*02*	*03*	*04*
≥ 65	7.2	7.3	6.7	8.8	7.9	8.1	6.5	8.2	7.9	6.6	6.2	7.0	7.4	6.4	7.1

< 65	92.8	92.7	93.3	91.2	92.1	91.9	93.5	91.8	92.1	93.4	93.8	93	92.6	93.6	92.9

The overwhelming majority of all visits (75% for patients ≥ 65 and 65% for those < 65) were between 7:00 and 17:59. Few in either group visited the PES between midnight and 06:59 (5% of visits for patients ≥ 65 and 10% of those < 65). The average number of daily visits from Monday to Friday was 16.3% ± 0.5 for those ≥ 65 and 15.3% ± 0.8 for those < 65 (NS, p = 0.055, *t *test for unpaired values). The average number of visits/month was equal (8.3%) for both groups (± 0.9, range 7.2 to 10.1 for patients ≥ 65, ± 0.5, range 7.8 to 9.1 for those < 65). No seasonal differences were observed between groups (*t*-test for unpaired values).

Frequency of use was examined by dividing patients into groups making one (N = 11,058), two or three (N = 2669), four to ten (N = 848) or eleven or more visits (N = 155). These anchor points have been shown to result in distinct diagnostic subgroups of PES users [10,23,29]. Age at the first visit from 1990 onward determined if patients were attributed a ≥ 65 or a < 65 tag. Within the ≥ 65 group single visits were made by 79.3% (N = 940), two to three visits by 16.5% (N = 196), four to ten visits by 3.9% (N = 46) and eleven or more by 0.3% (N = 3) patients. The corresponding numbers in patients < 65 were 74.7% (N = 10,118), 18.3% (N = 2473), 5.9% (N = 802) and 1.1% (N = 152), respectively. Overall, the frequent user profile was not significantly different between the two groups. However, the average number of visits/patient for all patients making over three visits, regardless of the total, was 5.5 (± 2.2) versus 7.7 (± 6.9) for those ≥ 65 and those < 65, respectively (p < 0.05, *t*-test for unpaired values).

### Socio demographic variables

Marital (N = 1332, N = 15,356, visits for patients ≥ 65 and < 65, respectively), employment (N = 1315, N = 16,632 visits for patients ≥ 65 and < 65, respectively) and residential profiles (N = 1392, N = 17,978 visits for patients ≥ 65 and < 65, respectively) were derived from the B dataset. The educational profile (N = 720 and N = 9,838 visits for patients ≥ 65 and < 65, respectively) was derived from the C dataset. All four profiles showed significant (Pchi2 & LRc2, *p *< 0.001) between group differences. Those ≥ 65 were more frequently widowed (28% versus 1%), less frequently single (24% versus 55%) than patients < 65 whereas the percent 'married/living together' (29 versus 23%) or 'separated/divorced' (20% each) were comparable. The proportion of patients ≥ 65 who were 'retired versus actively employed ' (82% versus 1.7%) differed markedly and expectedly from that of those < 65 (2.5% versus 32%,). Few patients ≥ 65 were receiving welfare, an income supplement program or any kind of employment insurance (11 versus 49% for those < 65). Those ≥ 65 were also more likely to live in a residence or another kind of non-family supervised housing (37.5% versus 14%), to be homeowners (14.5% versus 8.5%), less likely to be renting (apartment/room, 42.9% versus 64.8%) or living with family (1.5% versus 5.4%). Those ≥ 65 were less well educated, most having only a grade school (54% versus 11%) or high school (34% versus 56%) education, with fewer attaining either the college or university level (11% versus 33%).

### Arrival to the PES and prior clinical history

Type of arrival to the PES was taken from the C dataset (N = 1420, N = 18731 visits for patients ≥ 65 and < 65, respectively) and showed significant between group differences (Pchi2 & LRc2, *p *< 0.001). Patients ≥ 65 less frequently presented alone (14% versus 31%), being more often accompanied by a significant other or a caretaker (42% versus 30%). Police (5.7 versus 9.7%), ambulance (28 versus 21%), transfers from surrounding PESs (6.7 versus 6%) or "any other" (3 versus 2.6%) type of arrival was similar in those ≥ 65 and < 65. Voluntary (versus involuntary, regardless of the type of arrival) arrival did not differentiate one group from the other (81 and 83% of visits were voluntary for patients ≥ 65 and < 65, respectively).

Violence upon arrival was assessed from the B dataset (N = 1491, N = 19,379 visits, patients ≥ 65 and < 65, respectively) using logistic regression. Visits from patients ≥ 65 were less frequently tagged as violent (10 versus 20%, OR 0.55, *p *< 0.001, CI 0.47-0.64). No difference was found as to the nature of the aggressive acts (verbal, physical or both) but staff reactions to the acts did differ between the two groups (N = 67, N = 989 visits, patients ≥ 65 and < 65, respectively, Pchi2 & LRc2, *p *< 0.05). A verbal approach to contain the aggression was more often used with the elderly (66% versus 48%), rather than isolation (18% versus 23%), restraints alone (1% versus 7%) or the combination of restraints and isolation (15 versus 21%).

Both groups had a similar rate (about 40%) of at least one prior psychiatric hospitalization. Using logistic regression a history of substance abuse was less frequently found (OR 0.34, *p *< 0.001, CI 0.31-0.38) in patients ≥ 65 (27%, 457 of 1679 visits) than in those < 65 (57%, 13,937 of 24,371 visits). When present, substance abuse was primarily observed in men (versus women) in both groups (approximately 63% in men, 37% in women). The pattern of abuse was obtained for 333 (of the 457) visits and in 9206 (of the 13,937) visits of patients ≥ 65 and < 65, respectively. It was found to be much narrower in patients ≥ 65 (93% alcohol, 1% cannabis, 4% multiple substance, 1% benzodiazepines) versus (42% alcohol, 18% cannabis, 5% cocaine and 32% multiple substances, 3% other) in those < 65 (Pchi2 & LRc2, *p *< 0.001).

Current psychiatric medication (C dataset) was observed in 1135 of 1297 visits of patients ≥ 65 (88%) and in 12,420 of 17,353 visits (71%) of those < 65 (OR 2.6, *p *< 0.001, CI 2.2-3.0). The primary drug differentiating the former from those < 65 was the benzodiazepine class (39% versus 28%, OR 1.3, *p *< 0.001, CI 1.15-1.43).

### Visit characteristics

**Figure 1:**
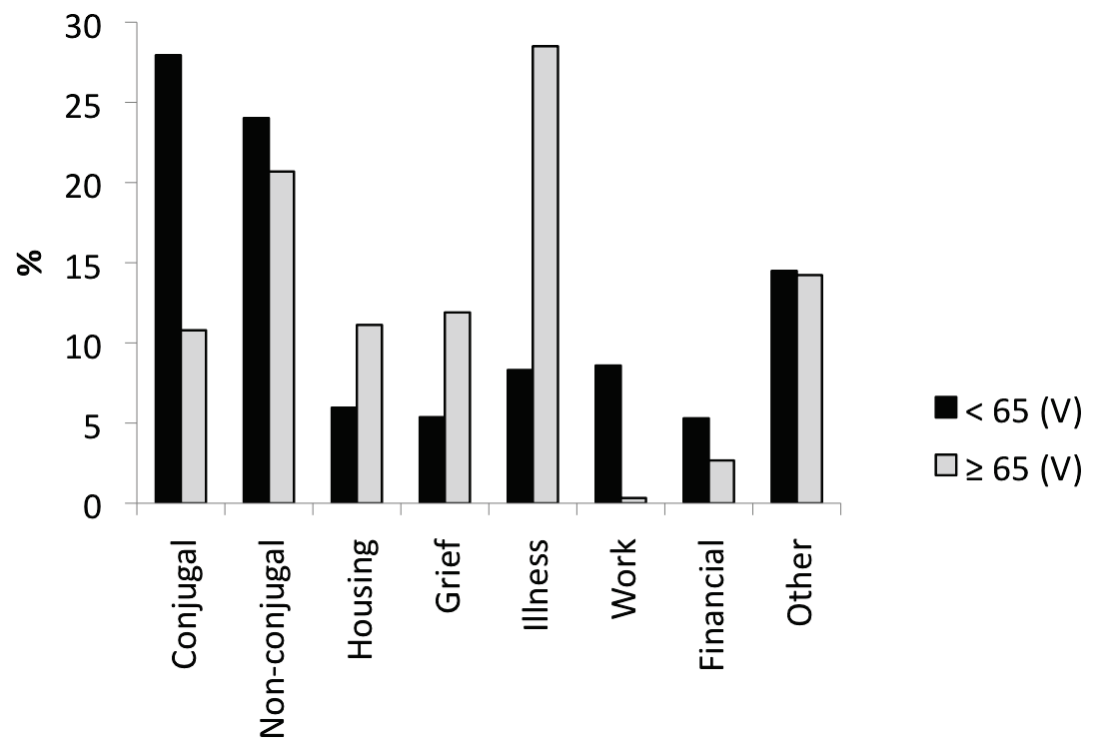
**Life-events associated with a PES visit (C dataset, N = 13,214 visits, patients < 65, N = 899 visits, patients ≥ 65)**. The profiles differed significantly (Pchi2 & LRc2, *p *< 0.001). Conjugal life events included those in married, common law or any long term intimate relationship. Non-conjugal included any other type of relationship. Illness was in "self" or in a "significant other".

**Figure 2:**
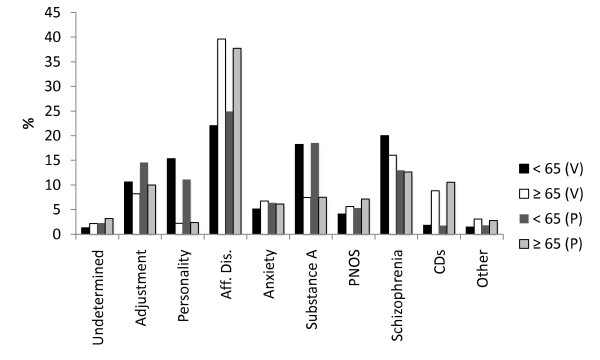
**Primary diagnostic profile for those ≥ 65 (visits = 2215, patients = 910) compared to those < 65 (visits = 29,704, patients = 7600), from the B dataset**. (V) = visits, (P) = patients. The profiles differed significantly, Pchi2 & LRc2, *p *< 0.001. Aff.Dis = unipolar, bipolar disorders and dysthymia, Substance A = alcohol and/or drugs, PNOS = psychosis not otherwise specified, CD = cognitive disorders, Other = impulse control, eating, sexual and primary sleep disorders.

**Figure 3:**
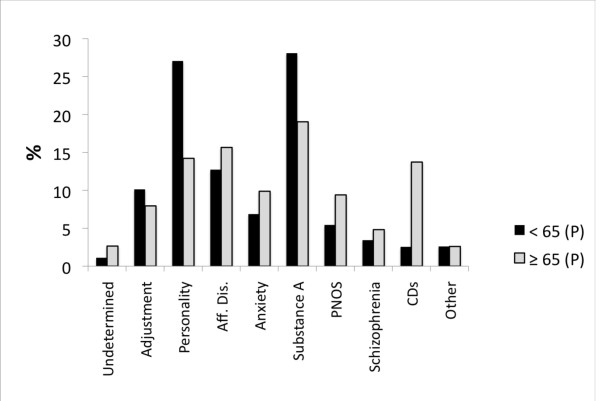
**The co-morbid diagnostic profile of patients ≥ 65 (N = 464) compared to that of patients < 65 (N = 8006), from the B dataset**. The profiles differed significantly, P <0.001, Pchi2 & LRc2, *p *< 0.001. For legend, see figure 2.

**Figure 4:**
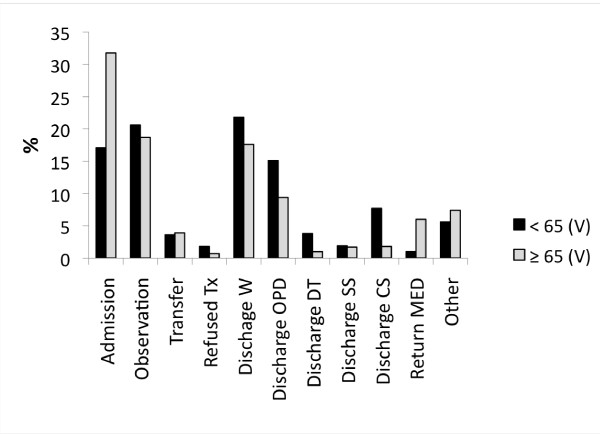
**Outcome profile of patients ≥ 65 (N = 3030) compared to that of patients < 65 (N = 39,455), from the combined A and B datasets**. The profiles differed significantly, Pchi2 & LRc2, *p *< 0.001. Admission = to a psychiatric ward, Observation = in the PES, Transfer = to another PES, Refused Tx = Left against medical advice, Discharge (W = without recommendations, OPD = to outpatient follow-up, DT = detoxification center, SS = social services, CS = crisis service), Return MED = patient was returned to the medical emergency service.

The over 30 reasons for a psychiatric referral were collapsed into 10 logical groupings (B dataset) for purposes of analysis. Compared to patients < 65 (27,286 visits), the profile for those ≥ 65 (2,132 visits) differed significantly (Pchi2 & LRc2, *p *< 0.001). It was marked by fewer referrals for "suicidal ideation" (8% versus 19%) or "suicide attempt" (2% versus 8%) and a greater number of referrals for "depression" (28 versus 20%). Few patients ≥ 65 were specifically referred for CDs (1.3%). Categories relating to "psychosis" (approximately 19% each), "hypomania/mania" (5 versus 3%), "anxiety" (11 versus 9%), "behavioral dyscontrol" (approximately 10% each), "other" (7 versus 6%) and "no specific reason" (7% each) were similar in both groups. About 65% of patients in both groups reported the presence of psychosocial stressors prior to their visit and these are illustrated in Figure 1. Diagnostic differences (for both visit and patient data) are presented in Figure 2. In addition, 30% for those ≥ 65 were co-morbid with a psychiatric disorder versus 42% of patients < 65. Figure 3 illustrates the most frequent psychiatric co-morbid diagnoses in both groups. Figure 4 illustrates visit outcomes.

The relationship between diagnosis and frequency of PES use is illustrated in table 2. In both groups, the proportion of patients with chronic psychosis increased and, those without a clear diagnosis, adjustment disorders or psychosis not otherwise specified, decreased with increasing frequency of use. Also, substance abuse contributed less to the overall profile of those with 11 or more visits. Notable between group differences were affective and personality disorders (increasing and decreasing, respectively, as frequency of use increased in those ≥ 65). The proportion with anxiety disorders was relatively independent of PES use in patients ≥ 65 but decreased with increasing visit frequency in patients < 65. For each frequency anchor point patients < 65 were significantly more co-morbid than those ≥ 65 (ORs ranging from 1.6 for single visits to 1.5 for 11 or more visits, P values < 0.01).

**Table 2:** Relationship between frequency^1 ^of PES use and diagnosis^2 ^(in %).

		*None*	*AD*	*PD*	*MAD*	*SCH*	*AX*	*SA*	*CD*	*PNOS*	*OTH*	*Visits^3^*
1 V 1 V	64- 65+	18 19	16 10	8 2	20 24	7 10	6 7	19 10	1 11	5 5	0.5 3	6314 557

2-3 V 2-3 V	64- 65+	9 12	11 5	10 1	21 33	14 14	5 5	21 7	2 7	4 6	2 10	4604 339

4-10 V 4-10 V	64- 65+	0 0	4 2	17 4	20 53	30 18	3 6	21 6	1 4	3 2	2 6	4994 428

11+ V 11+ V	64- 65+	0 0	0.5 0	25 0	15 48	45 35	1 7	12 5	1 5	1 0	0.5 0	5136 219

^1^Single visit (1 V), 2 to 3 visits (2-3 V), 4 to 10 visits (4-10 V) and 11 or more visits (11+ V).

^2^None = no clear diagnosis, AD = Adjustment disorders, PD = Personality disorders, MAD = Major affective disorders, SCH = Schizophrenia, AX = Anxiety disorders, SA = Substance abuse, CD = Cognitive disorders, PNOS = Psychosis not otherwise specified, OTH = Other (paranoid psychoses, eating disorders, impulse control...).

^3^Data taken from the combined A and C datasets. Totals for each row may not equal 100%, due to rounding.

Finally, as previously reported [30] staff were asked to qualitatively grade 1,604 visits from patients ≥ 65 and 21,607 visits of those < 65 (B dataset) for both pertinence and urgency. About 57% (≥ 65) and 52% (< 65) of visits were judged pertinent and urgent, 28% in both groups were judged pertinent but not urgent, 11% (≥ 65) and 15% (< 65) neither pertinent nor urgent. An assessment was not made in 5% in both groups. These profiles were not significantly different.

## Discussion

The median age of all citizens in the province of Quebec (Canada), of which Montreal metropolitan represents about half of the total population, increased by slightly more than 4 years between 1991 and 2001 and by 6 years between 1991 and 2006 [1,32]. This increase was mirrored by a 4-year increase in the median age of patients visiting our main PES site from January 1990 to August 2004.

In contrast to the reported increase in ED use for reasons of mental health by the elderly in the United-Sates from 1985 to 2000 [20], on a yearly basis, we found no proportional increase (in individual patients or their often multiple visits) in PES use during the over 14-year time course of our study. A relatively stable yearly rate of PES use, as assessed by the actual total number of visits/year by the elderly, was also reported by Cully et al., in Houston Texas, between 1994 and 2001 [12].

Many studies assessing PES use by the elderly suggest that they are underrepresented in this service [10-13]. In the present study they accounted for 7.2% of all PES visits, about half of their proportion in the surrounding population. Whether this implies that they actually underuse the PES is unclear. Despite ongoing research, a consensus as to what constitutes a "psychiatric emergency" remains elusive [30,33]. Qualitatively, visits made by the elderly were found to be indistinguishable from those made by younger patients, as assessed by the staff's subjective rating of each visit's pertinence and urgency. Over 50% and over 80% of their visits were tagged either "urgent" or "pertinent", respectively. However, many visits were also tagged "not urgent" in both the elderly and in those < 65. Using an "admission and/or observation" outcome as a less subjective index one might speculate that the elderly make better use of the PES than their younger counterparts (51% admission/observation rate versus 38% for those < 65). Indeed, a higher admission rate has been reported in several other studies [11-13,24,28].However, many non-psychiatric reasons, such as transportation problems or a poor social support network, may mitigate admitting an elderly, but not a younger, comparably ill patient.

Rather unexpected was the high number of repeat visits made by the elderly. High frequency PES use has typically been associated with a younger cohort, one with a diagnostic profile weighted towards schizophrenia and personality disorders [22,23,29,34,35]. In contrast, the elderly frequent user more typically suffered from an affective disorder, a finding in line with several studies showing a preponderance of affective and/or cognitive disorders in the overall elderly PES (and ED) diagnostic profile [11,12,24-28,36]. That only 10% of the elderly had CDs in our study is most likely attributable the ED staff's direct access to admission beds in a family medicine unit of the hospital specifically designated for the long term care and placement of CD patients. Overall, the elderly were less frequently co-morbid and the range of co-morbid diagnoses was much broader than that of younger patients, where personality and substance abuse disorders predominated. The lesser (and broader) co-morbidity may have contributed to the apparent under use of the PES by the elderly. On the whole though, the relative absence of substance abuse (history of and actual diagnosis) and schizophrenia's moderate contribution to the primary diagnostic profile is largely compatible with what has been previously reported [9,12,24,27]. When substance abuse was present alcohol was the overwhelming drug of choice, a finding that may prove useful in the future planning of specialized psychogereatric PESs.

Recently, it has been reported that about 30% of elderly and younger involuntary referrals to a southern California PES had positive urinary screens for drugs of abuse [37] and, if the screen had included alcohol, this number would have been much greater. In the present study about 7.5% and 19% of the elderly and 18.5% and 28% of patients < 65 had a primary or a co-morbid diagnosis of substance abuse (including alcohol), respectively. Several reasons may underlie the substantial differences between our results and those of Woo and Chen [37]. First, involuntary referrals may represent a specific subgroup that is more prone to substance misuse. In the present study, fewer than 20% of visits in both groups were involuntary. Second, our data is based upon a most probable (the most frequently attributed) primary and co-morbid diagnosis, not individual positive drug screens per visit. As such, in order to minimize diagnostic uncertainty we may have underestimated individual cases of substance misuse. For instance, in our study, a patient making 6 visits in which a personality disorder was diagnosed in 4 and substance abuse in 2, would receive a primary diagnosis of personality disorder with co-morbid substance abuse.

Most socio demographic variables differed in predictable ways. Many elderly were widowed and almost 40% were living in some type of supervised housing. Most were retired and 87% had at best a high school (or lower) level of education. Although they were by no means the picture of social and financially stability, those < 65 appeared more typical of the "downward drift" type patient often associated with the PES [10,22,34,38,39]. The latter were 75% single/divorced/separated, 49% either receiving welfare, unemployment insurance or some other type of supplementary income and 70% were either renters (rooms or apartments) or living with a family member. The life-events that brought the elderly to the PES were essentially age appropriate. They included illness (self or a significant other), grief reactions, housing and non-conjugal relational difficulties. These were reflected in the reasons noted by the ED medical staff in requesting a psychiatric evaluation as almost 40% pertained to depression or anxiety. Overall, with the above data would have predicted that the elderly would be less violent than younger patients and this was indeed the case.

A constellation of "core findings" typical of the elderly PES patient appears to be emerging. Underrepresentation, a preponderance of affective disorders, a higher admission rate, a gender difference, fewer self-referrals and medical conditions contributing to a PES visit and more frequent benzodiazepine use were found in the present, as well as several other studies [11-13,24,28]. About 6% of the elderly were returned to the ED for further medical investigation in our study, versus less than 1% for those < 65. To date, the few reports showing a predominance of men in the elderly visiting the PES are from services receiving a high proportion of police referrals [40,41]. Indeed, even in the present study, men predominated (56%) in police referrals of elderly patients (N = 50).

Our study suffers from several limitations. For instance, diagnostic validity and stability in a setting such as the PES. Bacca-Garcia et al., [42] using a retrospective semi administrative database found that validity and stability varied with diagnosis (best for schizophrenia, least for personality disorders) and setting (best in the inpatient, least in the outpatient, intermediate in the PES). Using purely administrative databases the PES has fared worse [43]. However, the prospective, non administrative nature of our database and the methodology used should have helped to reduce diagnostic uncertainty in a setting that has a much broader diagnostic range than the typical inpatient ward. If bias exists in our study it may be towards greater diagnostic stability and validity with increasing number of PES visits. Also, care must be taken when generalizing from what are largely regional data as they may not always accurately reflect national trends (such as the fact that the elderly with cognitive disorders were typically triaged to non-psychiatric services in this study).

## Conclusion

The elderly in the PES represent a more homogeneous group than their younger counterparts. This finding could be used at both the policy and clinical level to explore avenues that might be useful in increasing their quality of care. At a policy level, prevention may be an attainable objective. Proactive community support systems targeting those with newly diagnosed physical illnesses (or in a significant other) or grief reactions might be developed, as these stressors represent close to half of all stressors associated with a PES visit. At the local, organizational level developing increasingly efficient non-coercive verbal pacification measures to defuse potentially explosive situations would be pertinent, as well as triage systems with a particular focus on alcohol abuse, by far the substance of abuse of choice in this population. At a clinical level the elderly frequent user's affective disorders weighted diagnostic profile and lesser co-morbidity suggests that they may be more amenable to a shift towards more appropriate outpatient resources than frequent users < 65.

## List of abbreviations used

PES: Psychiatric Emergency Service; ED: Medical Emergency Department; CD: Cognitive Disorder; Aff.Dis: Affective disorders; Substance A: Substance abuse disorder; Patients 65 years of age and over: ≥ 65; Patients under 65 year of age: < 65; OR: Odds Ratio.

## Competing interests

The authors declare that they have no competing interests.

## Authors' contributions

YC was responsible for the design of the trial, data acquisition, data analysis and data interpretation. YC was also mainly responsible for the writing of the manuscript. MP, LB and EL were site-specific principal investigators with significant input as to the design of the database and full responsibility for its implementation at their respective sites. They also had significant input as to the interpretation of the results. All authors read and approved the final manuscript.

## Pre-publication history

The pre-publication history for this paper can be accessed here:

http://www.biomedcentral.com/1471-244X/11/111/prepub

## References

[B1] Statistics Canada Portrait of the canadian population in 2006, by age and sex http://www.statcan.gc.ca/bsolc/olc-cel/olc-cel?catno = 97-551-XWE2006001&lang=eng *Accessed at January 2008*

[B2] Regier DA Rae DS Narrow WE Kaelber CT Schatzberg AF Prevalence of anxiety disorders and their comorbidity with mood and addictive disorders Br J Psychiatry Suppl 1998 24 28 9829013

[B3] Bland RC Newman SC Orn H Prevalence of psychiatric disorders in the elderly in Edmonton Acta Psychiatr Scand Suppl 1988 338 57 63 3165596 10.1111/j.1600-0447.1988.tb08548.x

[B4] Streiner DL Cairney J Veldhuizen S The epidemiology of psychological problems in the elderly Can J Psychiatry 2006 51 185 191 16618010 10.1177/070674370605100309

[B5] Cainey J Coma LM Veldhuizen S Kurdyak P Streiner DL The social epidemiology of affective and anxiety disorders in later life in Canada Can J Psychiatry 2008 52 104 111 18357928 10.1177/070674370805300205

[B6] Wade TJ Cairney J Age and depression in a nationally representative sample of Canadians: a preliminary look at the National Population Health Survey Can J Public Health 1997 88 297 302 9401161 10.1007/BF03403892 PMC6990190

[B7] Martens PJ Fransoo R Burland E Burchill C Prior HJ Ekuma O Prevalence of mental illness and its impact on the use of home care and nursing homes: a population-based study of older adults in Manitoba Can J Psychiatry 2007 52 581 590 17953162 10.1177/070674370705200906

[B8] Sareen J Cox BJ Afifi TO Yu BN Stein MB Mental health service use in a nationally representative Canadian survey Can J Psychiatry 2005 50 753 761 16408523 10.1177/070674370505001204

[B9] Blixen C Lion J Psychiatric visits to general hospital clinics by elderly persons and younger adults Psychiatr Serv 1991 42 171 175 1997367 10.1176/ps.42.2.171

[B10] Chaput Y Lebel MJ Demographic and clinical profiles of patients who make multiple visits to psychiatric ermegency services Psychiatr Serv 2007 58 335 341 10.1176/appi.ps.58.3.335 17325106

[B11] Thienhaus OJ Rowe C Woellert P Hillard JR Geropsychiatric emergency services: utilization and outcome predictors Hosp Community Psychiatry 1988 39 1301 1305 3229754 10.1176/ps.39.12.1301

[B12] Cully JA Molinari VA Snow AL Burruss J Kotrla KJ Kunik ME Utilization of emergency center services by older adults with a psychiatric diagnosis Aging Ment Health 2005 9 172 176 10.1080/13607860412331336779 15804636

[B13] Woo BK Utilization patterns of psychiatric emergency services by elderly patients J Am Geriatr Soc 2009 57 182 183 10.1111/j.1532-5415.2009.02061.x 19170805

[B14] Strathdee SA Access to public mental health services among older adults with severe mental illness Int J Geriatr Psychiatry 2009 24 313 318 10.1002/gps.2123 18759380 PMC2645478

[B15] Depp CA Lindamer LA Folsom DP Gilmer T Hough RL Garcia P Jeste DV Differences in clinical features and mental health service use in bipolar disorder across the lifespan Am J Geriatr Psychiatry 2005 13 290 298 15845754 10.1176/appi.ajgp.13.4.290

[B16] Sewitch MJ Cole M McCusker J Ciampi A Dyachenko A Medication use and nonadherence to psychoactive medication for mental health problems by community-living canadian seniors with depression Can J Psychiatry 2008 53 609 620 18801224 10.1177/070674370805300908

[B17] Preville M Boyer R Grenier S Dube M Voyer P Punti R Baril MC Streiner DL Cairney J Brassard J The Epidemiology of Psychiatric Disorders in Quebec's Older Adult Population Can J Psychiatry 2008 53 822 832 19087480 10.1177/070674370805301208

[B18] Weinberger MI Mateo C Sirey JA Perceived barriers to mental health care and goal setting among depressed, community-dwelling older adults Patient Prefrence and Adherence 2009 3 145 149 10.2147/ppa.s5722 PMC2778418 19936156

[B19] Preville M Boyer R Vasiliadis HM Grenier S Voyer P Hudon C Streiner DL Cairney J Brassard J One-year incidence of psychiatric disorders in Quebec's older adult population Can J Psychiatry 2010 55 449 457 20704772 10.1177/070674371005500708

[B20] Larkin GL Claassen CA Emond JA Pelletier AJ Camargo CA Trends in U.S. emergency department visits for mental health conditions, 1992 to 2001 Psychiatr Serv 2005 56 671 677 10.1176/appi.ps.56.6.671 15939942

[B21] Currier GW Allen MH Organization and function of academic psychiatric emergency services Gen Hosp Psychiatry 2003 25 124 129 10.1016/S0163-8343(02)00287-6 12676426

[B22] Sullivan PF Bulik CM Forman SD Mezzich JE Characteristics of repeat users of a psychiatric emergency service Hosp Community Psychiatry 1993 44 376 380 8462947 10.1176/ps.44.4.376

[B23] Pasic J Russo J Roy-Byrne P High utilizers of psychiatric emergency services Psychiatric services (Washington, DC) 2005 56 678 684 15939943 10.1176/appi.ps.56.6.678

[B24] Waxman HM Carner EA Dubin W Klein M Geriatric psychiatry in the emergency department: characteristics of geriatric and non-geriatric admissions J Am Geriatr Soc 1982 30 427 432 7086021 10.1111/j.1532-5415.1982.tb03377.x

[B25] Shulman RW Marton P Fisher A Cohen C Characteristics of psychogeriatric patient visits to a general hospital emergency room Can J Psychiatry 1996 41 175 180 8722647 10.1177/070674379604100308

[B26] Coyne AC Gjertsen R Characteristics of older adults referred to a psychiatric emergency service J Ment Health Adm 1993 20 208 211 10.1007/BF02518689 10131296

[B27] Perez EL Blouin J Psychiatric emergency consultations to elderly patients in a Canadian general hospital J Am Geriatr Soc 1986 34 91 94 3944410 10.1111/j.1532-5415.1986.tb05474.x

[B28] Puryear D Lovitt R Miller D Characteristics of elderly persons seen in an urban psychiatric emergency room Psychiatr Serv 1991 42 802 807 1680098 10.1176/ps.42.8.802

[B29] Chaput YJ Lebel MJ An examination of the temporal and geographical patterns of psychiatric emergency service use by multiple visit patients as a means for their early detection BMC Psychiatry 2007 7 60 10.1186/1471-244X-7-60 17963530 PMC2190759

[B30] Chaput Y Paradis M Beaulieu L Labonte E A qualitative study of a psychiatric emergency Int J Ment Health Syst 2008 2 9 10.1186/1752-4458-2-9 18590555 PMC2499986

[B31] Quebec Statistics Institute(1) [Administrative regions, population by age and sex] http://www.stat.gouv.qc.ca/donstat/societe/demographie/dons_regnl/regional/index.htm Accessed at, *January *2008

[B32] Quebec Statistics Institute [Demographic summary of Quebec, 2008 edition] http://www.stat.gouv.qc.ca/publications/demograp/pdf2008/bilan2008.pdf *Accessed at January *2008

[B33] Claassen CA Hughes CW Gilfillan S McIntire D Roose A Lumpkin M Rush AJ Toward a redefinition of psychiatric emergency Health services research 2000 35 735 754 10966093 PMC1089145

[B34] Surles RC McGurrin MC Increased use of psychiatric emergency services by young chronic mentally ill patients Hosp Community Psychiatry 1987 38 401 405 3570189 10.1176/ps.38.4.401

[B35] Ledoux Y Minner P Occasional and frequent repeaters in a psychiatric emergency room Social psychiatry and psychiatric epidemiology 2006 41 115 121 10.1007/s00127-005-0010-6 16508721

[B36] Walker Z Leek CA D'ath PJ Katona CLE Psychiatric morbidit in elderly attenders at an accident and emergency department Int J Geriatr Psychiatry 1995 10 951 957 10.1002/gps.930101107

[B37] Woo BK Chen W Substance misuse among older patients in psychiatric emergency service Gen Hosp Psychiatry 2010 32 99 101 10.1016/j.genhosppsych.2009.08.002 20114135

[B38] Arfken C Zeman LL Yeager L Mischel E Amirsadri A Frequent visitors to psychiatric emergency services: staff attitudes and temporal patterns J Behav Health Serv Res 2002 29 490 496 10.1007/BF02287355 12404943

[B39] Oyewumi LK Odejide O Kazarian SS Psychiatric emergency services in a Canadian city: I. Prevalence and patterns of use Can J Psychiatry 1992 37 91 95 1562965 10.1177/070674379203700203

[B40] Niizato K Suzuki M Kawada F Oshima K Matsushita M Analysis of psychogeriatric patients in the psychiatric emergency system Psychogeriatrics 2003 3 109 114 10.1111/j.1479-8301.2003.00018.x

[B41] Sawayama E Takahashi M Arai H Nakajima K Kano A Sawayama T Miyaoka H Characteristics of elderly people using the psychiatric emergency system Psychiatry Clin Neurosci 2009 63 577 579 10.1111/j.1440-1819.2009.01975.x 19457212

[B42] Baca-Garcia E Perez-Rodriguez MM Basurte-Villamor I Del Moral AL Jimenez-Arriero MA De Rivera JL Saiz-Ruiz J Oquendo MA Diagnostic stability of psychiatric disorders in clinical practice Br J Psychiatry 2007 190 210 216 10.1192/bjp.bp.106.024026 17329740

[B43] Folsom DP Lindamer L Montross LP Hawthorne W Golshan S Hough R Shale J Jeste DV Diagnostic variability for schizophrenia and major depression in a large public mental health care system dataset Psychiatry Res 2006 144 167 175 10.1016/j.psychres.2005.12.002 16979244

